# Comment on “Heterogeneity in PD-L1 expression in malignant peritoneal mesothelioma with systemic or intraperitoneal chemotherapy”

**DOI:** 10.1038/s41416-020-01214-8

**Published:** 2020-12-18

**Authors:** Haitang Yang, Sean R. R. Hall, Beibei Sun, Feng Yao

**Affiliations:** 1 Department of Thoracic Surgery, Shanghai Chest Hospital, Shanghai Jiao Tong University, 200030 Shanghai, People’s Republic of China; 2 Gillies McIndoe Research Institute, Wellington, 6021 New Zealand; 3 Institute for Thoracic Oncology, Shanghai Chest Hospital, Shanghai Jiao Tong University, 200030 Shanghai, People’s Republic of China

**Keywords:** Mesothelioma, Tumour immunology

Immunotherapy has revolutionised the treatment of various cancers. Programmed death-ligand 1 (PD-L1) expression is a clinically approved biomarker to predict benefit from immune checkpoint blockade (ICB). However, the response to ICB is highly heterogeneous, indicating that additional biomarkers are urgently needed to guide the precise management of immunotherapy for cancer patients.

Unlike other cancer types, immunotherapy lags significantly in the field of malignant pleural or peritoneal mesothelioma (MPM) patients, a lethal malignancy characterised by frequent inactivation of tumour-suppressor genes (TSGs), e.g., *BAP1*, *NF2*, *LATS1/2*, and *TP53*.^1,2^ Recently, White et al. conducted a retrospective analysis of PD-L1 expression in malignant peritoneal mesothelioma who received systemic or intraperitoneal chemotherapy.^3^ Their findings identified higher levels of PD-L1 expression related to samples with germline mutations (20 vs. 1%, *p* = 0.04) and higher somatic mutational burdens. Interestingly, in the biopsies before systemic chemotherapy, they observed a significantly lower PD-L1 expression (6 vs. 16%, *p* = 0.02). Notably, among the 20 (38%) out of 53 samples with measured PD-L1 expression before and after chemotherapy (systemic or intraperitoneal), 7 (35%) samples had <1% staining before and after treatment, with PD-L1 upregulation observed in 6 (30%) but downregulation in 8 (40%) samples after chemotherapy. Their data highlight the high heterogeneity in PD-L1 expression between matched samples before and after chemotherapy, which may provide critical implications for clinical management of ICBs.

The evidence provided by White and colleagues may explain the failure of a recent phase III clinical trial conducted by Popat et al., evaluating the efficacy of anti-PD-1 immunotherapy (pembrolizumab, *n* = 73) vs. chemotherapy (gemcitabine or vinorelbine, *n* = 71) in relapsed MPM, and patients were unselected for PD-L1 status.^4^ Unfortunately, there is no improvement for progression-free survival and overall survival (OS) for pembrolizumab over chemotherapy.

Given the above evidence, several factors need to be considered for the further design of immunotherapy for MPM. Recent evidence has suggested high heterogeneity of MPM tumours and the importance to optimise histological and molecular classifications for improved immunotherapies.^5–7^

First, different histological subtypes are associated with differential tumour immune microenvironment (TIME), particularly T cell repertoire that plays critical roles in determining the antitumour immunity.^2,7^ Specifically, compared to other histological subtypes, the sarcomatoid subtype is enriched with T cells infiltration and associated with higher PD-L1 expression,^2,7^ a clinically approved biomarker to predict benefit from ICB. We note that, in the White et al. study, the sarcomatoid subtype was not studied; however, it is interesting to speculate that this tumour subtype might respond better to ICB.

Second, MPM is predominantly characterised by loss of functions in TSGs, which leads to heterogeneous molecular aberrations.^6^ Our recent evidence has demonstrated that deficiency in the key Hippo pathway core component LATS1/2, rather than other major TSGs, is significantly correlated with enriched TIME and high PD-L1 expression, suggesting a rationale for ICB therapy for this subset.^6^ Encouragingly, retrospective analysis of patients after ICBs showed that patients with mutated *LATS1/2* have significantly longer OS than patients with wild-type *LATS1/2*.^6^ More importantly, based on high-throughput correlation analysis of drug sensitivity profiling with gene expression, the evidence from our study points out the potential of dasatinib to modulate antitumour immunity. Of particular interest, we also showed that dasatinib selectively impairs *LATS2*-mutant MPM cells, compared with other MPM cells carrying mutations in other TSGs. Our findings suggest that dasatinib may represent a promising therapeutic for *LATS2*-mutant MPM, which might affect both tumour cells and TIME. Supporting this notion, Chen et al.^5^ recently evaluated the evolution of T cell repertoire heterogeneity of MPM under dasatinib treatment, demonstrating that, compared to pretreatment tumours, dasatinib treatment induced a significant increase in T cell clonality, despite the presence of highly heterogeneous T cell receptor repertoire within MPM samples. The findings by Chen and colleagues highlighted the combination of ICBs with dasatinib as a promising treatment for a subset of MPM.

Besides, pathway dysregulation might also affect the PD-L1 expression and therapeutic response. We mined the reverse-phase protein array (quantifying 220 proteins including PD-L1) data of The Cancer Genome Atlas MPM cohort, which revealed several proteins that are significantly correlated, positively or negatively, with the PD-L1 protein level (Fig. 1), particularly proteins that negatively regulate the AKT-mammalian target of rapamycin (mTOR) signalling pathway, e.g. PTEN, TUBERIN, and 4EBP1_pT37T46. Intriguingly, dysregulated mTOR pathway has been highlighted in clinical MPM tumours.^2^

**Fig. 1:**
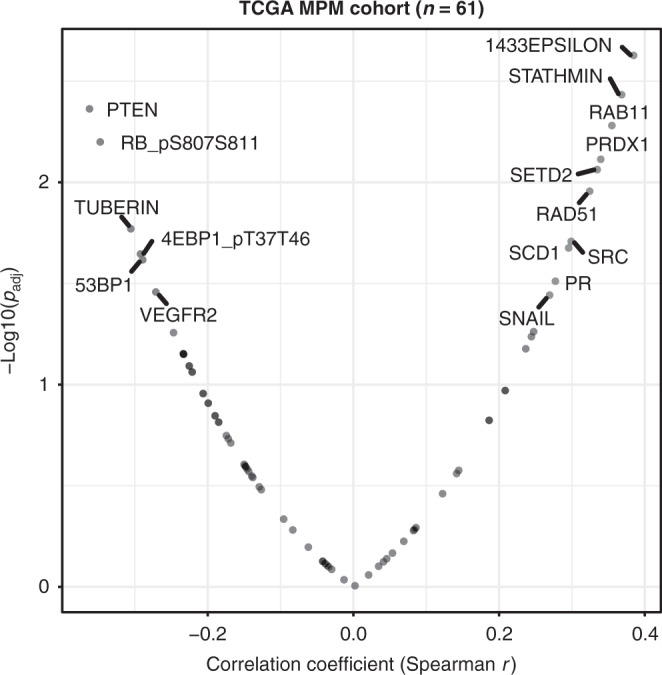
Dysregulated pathways potentially affect the PD-L1 expression. Proteins correlated with PD-L1 expression across the TCGA (The Cancer Genome Atlas Program) MPM cohort (*n* = 61). Blue dots indicate the significantly negatively correlated genes while the red the positively correlated ones. Normalised level 4 data of reverse phase protein array (RPPA) were downloaded from The Cancer Proteome Atlas (TCPA) database (https://tcpaportal.org/tcpa/).

Last, the spatial heterogeneity in the expression of PD-L1^8–10^ may influence its role as a predictive biomarker for therapeutic response to ICBs.

Overall, multiple factors, such as histology and TSGs, may affect the PD-L1 expression in MPM.

## Data Availability

Not applicable.
